# The relationship between context, structure, and processes with outcomes of 6 regional diabetes networks in Europe

**DOI:** 10.1371/journal.pone.0192599

**Published:** 2018-02-15

**Authors:** Mahdi Mahdavi, Jan Vissers, Sylvia Elkhuizen, Mattees van Dijk, Antero Vanhala, Eleftheria Karampli, Raquel Faubel, Paul Forte, Elena Coroian, Joris van de Klundert

**Affiliations:** 1 National Institute of Health Research, Tehran University of Medical Sciences, Tehran, Iran; 2 Institute of Health Policy and Management, Erasmus University Rotterdam, Rotterdam, The Netherlands; 3 Hook of Holland, Rotterdam, The Netherlands; 4 HEMA Institute, Aalto University, Aalto, Finland; 5 National School of Public Health, Alexandras, Athens, Greece; 6 Joint Research Unit in ICT applied to Healthcare Process Re-engineering (eRDSS), Valencia, Spain; 7 University of Valencia, Valencia, Spain; 8 The Balance of Care Group, London, United Kingdom; 9 Institute for Learning Innovation, Friedrich-Alexander-University Nuremberg-Erlangen, Nuremberg, Germany; The Chinese University of Hong Kong, HONG KONG

## Abstract

**Background:**

While health service provisioning for the chronic condition Type 2 Diabetes (T2D) often involves a network of organisations and professionals, most evidence on the relationships between the structures and processes of service provisioning and the outcomes considers single organisations or solo practitioners. Extending Donabedian’s Structure-Process-Outcome (SPO) model, we investigate how differences in quality of life, effective coverage of diabetes, and service satisfaction are associated with differences in the structures, processes, and context of T2D services in six regions in Finland, Germany, Greece, Netherlands, Spain, and UK.

**Methods:**

Data collection consisted of: a) systematic modelling of provider network’s structures and processes, and b) a cross-sectional survey of patient reported outcomes and other information. The survey resulted in data from 1459 T2D patients, during 2011–2012. Stepwise linear regression models were used to identify how independent cumulative proportion of variance in quality of life and service satisfaction are related to differences in context, structure and process. The selected context, structure and process variables are based on Donabedian’s SPO model, a service quality research instrument (SERVQUAL), and previous organization and professional level evidence. Additional analysis deepens the possible bidirectional relation between outcomes and processes.

**Results:**

The regression models explain 44% of variance in service satisfaction, mostly by structure and process variables (such as human resource use and the SERVQUAL dimensions). The models explained 23% of variance in quality of life between the networks, much of which is related to contextual variables. Our results suggest that effectiveness of A1c control is negatively correlated with process variables such as total hours of care provided per year and cost of services per year.

**Conclusions:**

While the selected structure and process variables explain much of the variance in service satisfaction, this is less the case for quality of life. Moreover, it appears that the effect of the clinical outcome A1c control on processes is stronger than the other way around, as poorer control seems to relate to more service use, and higher cost. The standardized operational models used in this research prove to form a basis for expanding the network level evidence base for effective T2D service provisioning.

## Introduction

Diabetes is amongst the leading causes of morbidity around the world. The prevalence of diabetes amounted to 435 million in 2015 globally [1], of which around 90% was Type 2 Diabetes (T2D) [2]. In Europe, 55.4 million of individuals 20 years or older have T2D requiring more than 100 billion USD for treatment [1].

Over the course of the chronic condition T2D, the services delivery to a patient typically involve multiple provider organisations at various locations in the region in which the patient lives [3, 4]. Below, we will refer to such regional collections of provider organisations as T2D networks. T2D networks can be formally and explicitly established, e.g. through contracts between organisations, or through public administration in a public health sector. Alternatively, a T2D network can be defined implicitly as the collection of health service provider organisations jointly visited by the population of T2D patients in a region. Either way, clinical outcomes (such as A1c level), functional outcomes (such as quality of life), and experience outcomes (such as service satisfaction) depend to a large extent on the joint performance of the organisations in the T2D network [5].

**Fig 1:**
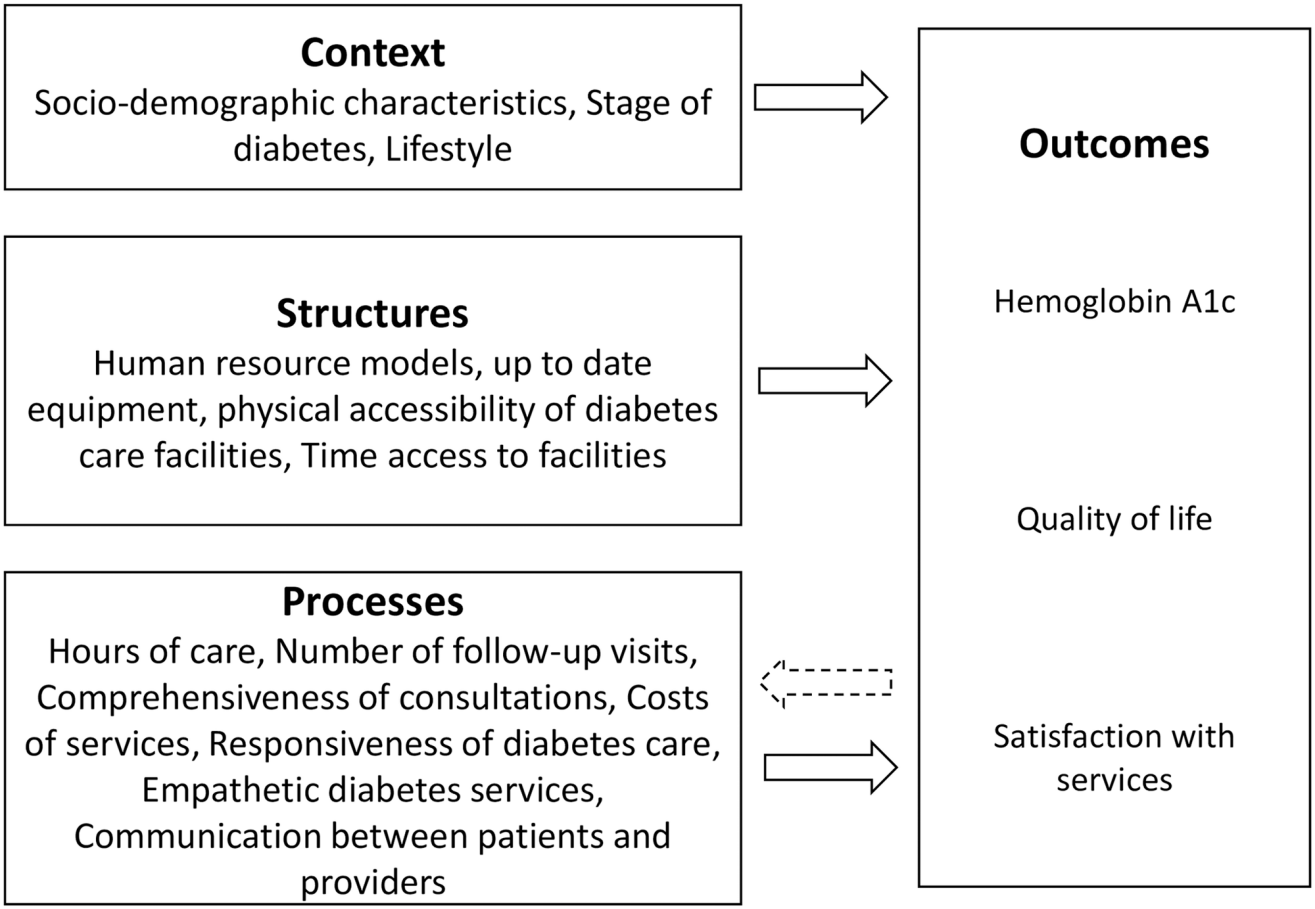
The extended Structure-Process-Outcome (SPO) model proposed for T2D networks.

Previous studies have indeed shown that these outcomes vary among T2D networks [6–9]. A seminal and often applied conceptual framework to develop understanding of factors that explain outcomes is the Structure-Process-Outcome (SPO) model proposed by Donabedian [10]. First, *structures* refer to attributes of settings where services are provided, which include facilities and equipment, human resources, and policies. Second, *processes* refer to attributes (such as completeness, continuity, functional quality) of activities for diagnosis and treatment. The SPO model posits that appropriate structures facilitate service processes that are more likely to result in desired (high quality) outcomes. Additionally, one may expect that the outcomes produced in a T2D network affect the health service use, i.e. the processes [6]. Moreover, when considering regional T2D networks, one should take into account that the regional context, other than structures, (e.g. geography or demography) also impacts the outcomes. The full conceptual model that we propose for studying T2D networks is therefore as depicted in Fig 1.

Systematic literature review has revealed that very few studies consider how outcomes relate to service structures and processes within the contexts defined by regional T2D networks [11]. The research question that we address therefore is: How do structures, processes, and context of regional provider networks relate to T2D outcomes? As our main interest is to advance understanding of the network design, we will be particularly interested in the network structures and processes, and consider context predominantly as a set of covariates.

While this question has received little attention for T2D networks, there is evidence at the levels of provider organisations and (solo) practitioners within these networks. Collins et al. state that the quality of life of T2D patients varies with the structure of organisations [12]. In fact, structures which more heavily use nurses–and therefore make less use of GPs and/or medical specialists—have been associated with higher service satisfaction [13]. Other resources, such as equipment, are known to impact outcomes as well [14]. Moreover, structures which improve access to care have been reported to positively affect service satisfaction and the health state of T2D patients [15, 16].

Process characteristics which have been investigated for single provider organisations and solo practitioners in relation to outcomes of T2D care include frequency, duration, and comprehensiveness of services for both diagnosis and treatment [17]. Moreover, patient reported process characteristics such as perceived waiting time, responsiveness, and empathy have been considered in relation to service satisfaction [18, 19]. Furthermore, evidence suggests a relationship between service frequency and health outcomes [20].

Given the complexity of regional T2D provider networks, it is likely that outcomes relate to structures, processes, and context in complex and interacting ways. Given the lack of evidence on these relationships we aim to contribute to an initial evidence base by exploring independent relationships of structures, processes, and context with outcomes. Primarily, we assess the independent cumulative proportion of variance in service satisfaction and quality of life by structure, process, and context variables. The main approach will be to verify whether the evidence found for solo practitioners and/or single provider organisations extends to the T2D network level.

The importance of developing understanding on these relationships not only stems from the scientific relevance of building an evidence base that considers the network level, but also from the societal need to effectively and affordably address the ever more prevalent condition of T2D.

This manuscript builds on the EU FP7 project MANAGED OUTCOMES, which aimed to develop such understanding for a variety of highly prevalent conditions [21]. All data collection and modelling was funded through MANAGED OUTCOMES.

## Materials and methods

### Study settings

Within MANAGED OUTCOMES, we conducted a cross sectional study involving T2D networks and patients from six regions: Keski-Suomi (Finland); Bamberg (Germany); Herakleion (Greece); NieuweWaterwegNoord & DelftWestlandOostland (NWN & DWO) (The Netherlands); Valencia (Spain); and Tower Hamlets (United Kingdom). S1 Appendix provides detailed background information on the regions.

The network structures and processes were modelled using the MANAGED OUTCOMES network model, depicted in S1 Framework as well. Many of the variables in this model describe features of the network as a whole. Likewise, some of the context and outcome variables regard the (whole) population of T2D patients living in the region. Other variables however, regard individual patients. Hence, below we speak of network level variables (and network level data) and patient level variables (and patient level data).

The patient population studied consists of the individuals diagnosed with T2D registered and serviced by primary care providers in the regions. While there is no requirement that patients are exclusively serviced by primary care providers, we have excluded patients primarily treated in secondary or tertiary care. Individuals in a pre-diabetes stage are also not considered.

Below, we first specify the variables included in the analysis, and then further outline the research design and analysis methods.

### Ethics statement

The study was approved in each country involved in the study. The Keski Suomi study was approved by the Ethics Committee of the Central Finland Health Care District. The Bamberg study was approved by the Ethics Committee of the Medical Faculty of the Friedrich-Alexander University in Erlangen-Nürnberg. The Herakleion study was approved by the Scientific Committee of the hospital in Herakleion. The NieuweWaterwegNoord & DelftWestlandOostland study was approved by the board of directors of the Primary Care Group ZEL. The Valencia study was approved by the Hospital La Fe Ethical Committee. The Tower Hamlets study was approved by the NHS National Research Ethics Service. Permission for use of data was received from the Ethics Committee of the Central Finland Health Care District (statistical data at aggregate level), the Ethics Committee of the Medical Faculty of the Friedrich-Alexander University in Erlangen-Nürnberg (statistical data at aggregate level), the Scientific Committee of the hospital in Herakleion (statistical data and access to patient records), the Scientific Council of the IPCI system of the department of Medical Information of the Erasmus Medical Centre (statistical data at aggregate level), the Hospital La Fe Ethical Committee (statistical data at aggregate level) and the NHS National Research Ethics Service (statistical data and access of patient records through the clinicians of the local diabetes research network).

### Variables

#### Outcomes

The analysis includes three commonly investigated outcome variables representing the three domains clinical outcomes, functional outcomes, and experiential outcomes. The clinical outcome variable is effective coverage of diabetes care, the functional outcome is quality of life, and the experiential outcome is service satisfaction.

Quality of life was measured in terms of EQ5D at patient level through the five dimensions: mobility, self-care, usual activity, pain/discomfort, and anxiety/depression [22]. On each dimension, valid responses for items were: having no problems, having some or moderate problems, being unable to do/having extreme problems. The Utility index score of EQ5D per subject was calculated from all five dimensions using the Dolan Utility Index [23].

Service satisfaction was measured at the patient level as the overall/global satisfaction with the T2D network service performance on a Likert scale from 1 (extremely dissatisfied) to 7 (extremely satisfied). For the purpose of comparison, it was subsequently rescaled to a percentage (0% for lowest satisfaction to 100% for highest).

The effective coverage percentage is defined as the percentage of T2D patients for which service provisioning has resulted in an A1c level below 53 mmol/mol [24, 25]. For feasibility of data collection, the clinical outcome variable A1c was not considered at patient level, but at network level.

#### Structure

Following our conceptual framework and the evidence reviewed in the introduction, we considered structural characteristics regarding resources—both human resources and equipment—and accessibility of the locations [10]. The human resources considered included practice nurses, diabetes nurses, physicians, and specialists. More specifically, we distinguished three human resource models: 1) a model in which medical specialists played a leading role in the service delivery, 2) a model in which the GP/Family doctor played a central role, and 3) a model in which a nurse plays a central role (henceforth referred to as the nurse-based model) [13, 26]. Per region, these models don't differ from patient to patient, and hence these models apply to the network level.

The resource concept of equipment is operationalized through the (patient reported) service quality dimension ‘tangibles’ from the SERVQUAL model [18, 19]. Accessibility of locations is measured in terms of physical distance (between patient home and service facilities) and in terms of travel time (between patient home and service facilities). Both variables are measured at patient level.

#### Process

Process variables were also selected based on our conceptual model and earlier evidence for single provider organization studies and solo practitioner studies as mentioned in the introduction. We included average annual hours of care provided per patient, average annual cost per patient, annual number of follow up visits, comprehensiveness of consultation, outpatient waiting time, and a selection of reportedly most influential SERVQUAL variables.

Hours of care are measured at the network level. More specifically, it measures the average hours of care at network level by summing over all patients, the time the delivered service activities take per year. Based on these breakdowns of service processes into activities, average cost per patient are calculated by calculating activity based costs for these activities as on average delivered to patients [27]. Costs are adjusted by purchasing power parity (PPP) for 2011 [28].

The annual number of visits is measured at the patient level, as is the case for comprehensiveness of consultation (referring to the degree to which all questions of the patient are addressed and discussed within a single consultation visit), and outpatient waiting time.

The patient level SERVQUAL process variables included are responsiveness, timeliness, empathy, caring, and communication [29], so as to understand the relationship between well-established service process quality determinants and the outcome service satisfaction [30, 31].

#### Context

Contextual characteristics included demographic aspects of the T2D patient population. Following our conceptual framework and earlier evidence on T2D services, we included the patient level variables age, gender, education, time since diagnosis of T2D, stage of T2D [32, 33], and patient behavior [34].

Patients were classified to either have minimum required education, or higher. Patient behavior included smoking, physical activity habits, and knowledge of A1c value. Patients were either current smoker or past/non-smoker. Physical activity is measured as the number of days the patient has at least 30 minutes of moderate or intensive physical activity, average over the last four weeks (at time of survey).

To operationalize stage of T2D, we distinguished three segments of T2D diabetes patients:

1. T2D diagnosed, treatment is mainly in primary care and consists of life style intervention (lifestyle segment),
2. T2D diagnosed, treatment is mainly in primary care and consists of life style intervention in combination with oral diabetic drugs (medication segment),
3. T2D diagnosed, treatment is mainly in primary care and consists of life style intervention, oral drugs, and/or insulin injections (insulin segment).

### Data collection

Data collection took place in 2011–2012. Network level data mostly originate from administrative databases, and were collected using the structured operational model templates developed in MANAGED OUTCOMES, as described in S2 Appendix. As an example, S3 Appendix provides a description of the network level data for the Dutch network NWN & DWO.

Patient level data were collected by patient survey. Over the six regions, 5972 questionnaires were distributed to patients, predominantly by mail. Except for number of follow up visits (which had a one-year recall period), all variables had a one-month recall period. Provider organisations within the T2D networks assisted in selecting survey respondents.

### Analysis methods

Differences between the networks with regard to EQ5D and service satisfaction were analysed using analysis of variance (ANOVA), Levene’s test for equality of variance, and Welch F test [35]. Comparison between the countries with regard to effective coverage percentage was performed using Chi-square test.

As a main analysis model, we consider how variance in patient level outcomes relates to structure, process, and context variables. Analysis methods apply to the outcome variables service satisfaction and quality of life. The analysis considers 3 models for the outcomes service satisfaction and quality of life. Model 1 only includes context variables which we consider as covariates henceforth. Model 2 includes context and structure variables. Model 3 includes context, structure and process variables. These models are built using linear regression models. For each model, we report R^2^ and significance of change in R^2^ to define statistically significant increase in the proportion of explained variance in the outcomes. The relationship between the context, structure and process variables with outcomes is also determined by its coefficient value.

The analysis for the outcome measure effective coverage percentage necessarily uses a different method as this variable is measured at the network level. We inspect the relationships graphically, especially in relation to the process measures hours of care and costs, for which we conjecture that the relationship is bidirectional.

To minimize exclusion of cases from the regression model attributable to missing values we used imputation. Except for the missing values of EQ5D, which were imputed differently, the missing values were imputed using multiple imputations using a default Markov Chain Monte Carlo method. Imputation was iterated five times which thus produced five datasets [36]. Missing values of EQ5D dimensions are imputed only if at most one dimension was missing. Statistical analyses were performed using the IBM SPSS statistical package version 20.

## Results

Out of the total of 5972 questionnaires distributed in the six regions, 1638 were returned. The 179 questionnaires returned by respondents who indicated inability to master the language of the country in which they lived were excluded, resulting in a total of 1459 participants to be included in the analyses (a 24.4% response rate). Response rates ranged from 61.9% in Germany to 15.5% in the UK. The percentage of missing values, excluding EQ5D dimensions, was on average 9.1%. The missing values ranged from 2.0% for smoking to 17.1% for physical activities.

S4 Appendix includes a summary of the contextual differences between the patient populations from the six regions with regard to age, gender, and disease stage. Network level data were collected in all regions, except for Bamberg, Germany.

### Differences in outcomes between provider networks

Descriptive statistics on the outcome variables effective coverage, EQ5D, and service satisfaction from the six regional T2D networks are presented in Table 1. After briefly reviewing the outcome data, we firstly present the main analysis results on the outcome variables service satisfaction and EQ5D, and subsequently, the coarser results on effective coverage percentage.

**Table 1:** Outcome variables for the six regional provider networks and analysis of variance.

	Keski-Suomi (FI)		Bamberg (GE)	Hera kleion (GR)	NWN & DWO (NL)	Valencia (SP)	Tower Hamlets (UK)	Total	Welch F^1^	Df1	Df2
**Patients with A1c<53 mmol/mol**	77		NA	42	72	60	56	61			
**EQ-5D utility** ^2^											
**Mean**		0.81	0.76	0.70	0.84	0.74	0.69	0.77	25.1*	5	514.4
**SD**		0.17	0.2	0.2	0.17	0.24	0.27	0.22			
**95% CI**^3^		0.78	0.74	0.67	0.83	0.7	0.66	0.75			
		0.83	0.79	0.73	0.86	0.79	0.72	0.78			
**Min**		0.09	0.03	-0.08	0.2	0.03	-0.59	-0.59			
**Max**		1	1	1	1	1	1	1			
**N**		181	271	174	379	110	304	1419			
**Services satisfaction**^4^											
**Mean**		86.82	79.33	70.81	86.25	69.18	77.38	79.84	18.5*	5	494.7
**SD**		19.01	24.58	26.15	20.36	26.71	25.46	24.22			
**95% CI**^3^		83.96	76.31	66.84	84.14	64.04	74.46	78.55			
		89.68	82.34	74.78	88.36	74.33	80.3	81.13			
**Min**		0	0	0	0	0	0	0			
**Max**		100	100	100	100	100	100	100			
**N**		172	258	169	360	106	294	1359			

Note:

* p<.05

** p<.01

*** p<.001

^1^ Welch F test is reported as differences in variance between regions are significant.

^2^ EQ-5D-utility is calculated according to Dolan. This scale is standardized with 0 for death and 1 for full health. Missing values of EQ5D dimensions are imputed only if at most one dimension was missing.

^3^ 95% CI = 95% confidence interval (the first line shows lower bound and the second line shows upper bound).

^4^ This scale is standardized from 0 to 100.

Keski-Suomi has the largest number of patients with effective coverage (77%), followed by NWN & DWO. Herakleion has the lowest level of this outcome (42%). Valencia and Tower Hamlets are in between with 60% and 56% respectively. From a Chi-square test, we conclude that the levels of effective coverage significantly differ between these regions (p<0.001).

EQ5D varies significantly among regions. Average EQ5D was highest in NWN&DWO in Netherlands and lowest in the UK region Tower Hamlets. Service satisfaction significantly varies across regions as well. Valencia has the lowest service satisfaction, whereas the service satisfaction of the patient population Keski-Suomi stands out positively.

### Relationships between structures and processes with service satisfaction

The relationships between structure and process variables with service satisfaction are given in Table 2. Model 1, which only includes context variables explains up to 3% of variance in service satisfaction. Model 2, which includes context and structure variables, explains slightly less than one third of the variance in satisfaction (R^2^ = .31, p<.001). The change in the amount of explained variance between Models 1 and 2 is highly significant (R^2^ Change = . 27-.28, p<.001). Model 3, which includes context, structure, and process variables, explains up to 44% of variance in satisfaction. Again, the change in the amount of explained variance compared to Model 2 is highly significant (R^2^ Change = .12-.14, p<.001). Of the context variables, education is significantly and positively related to service satisfaction in Models 1 and 2, but only gender is significant in Model 3. Male patients are on average significantly more satisfied.

**Table 2:** Regression analysis of satisfaction with services ^a^.

		Model 1	Model 2	Model 3
		β	β	β
**Covariates**	Age	.023	.010	.014
	Gender			
	- Female (reference)	0	0	0
	- Male	2.849	3.806*	3.594*
	Education			
	- Minimum school leaving age (reference)	0	0	0
	- More than minimum school leaving age	4.582*	2.715*	.934
	Time since diagnosis	-.003	-.020	-.042
	Stage of diabetes			
	- Lifestyle segment b	0	0	0
	- Medication segment c	-.652	-.108	-.778
	- Insulin injection segment d	.071	-.438	-2.099
	Drink			
	- Non-drinker (reference)	0	0	0
	- Drinker	2.105	.354	-.997
	Smoke			
	- Former smoker/non-smoker (reference)	.000	0	0
	- Current smoker	-3.420	-3.489	-3.121
	Physical activity	.200	.230*	.077
	Knowledge of A1c			
	- Unknown value of A1c (reference)	0	0	0
	- Known value of A1c	-.752	.749	-.249
**Structure**	Human resource model			
	- GP/internist model (reference)		0	0
	- GP/family doctor model		-1.569	-.884
	- Nurse-based model		6.325*	2.964
	Equipment		7.522*	2.285*
	Travelling distance to facility		.051	.002
	Travelling time to facility		.050	.100
**Process**	Number of follow up visits			.150
	Comprehensiveness of follow up visits			2.423*
	Waiting time in facility			-.098*
	Timeliness			1.717*
	Responsiveness			3.139*
	Empathy			-1.718*
	Caring			3.135
	Communication			.758
	R^2^ Change		.27-.28	.12-.14
	R^2^	.02-.03	.29-.31	.42-.44
	F Change	2.90–3.89	93.24–102.67	32.50–38.87
	df1	10	5	8
	df2	1247	1242	1234
	Sig of F change	0.001	0.000	0.000

^a^ Service satisfaction measured on a scale ranging from 0 to 100. Unstandardized coefficients,

*p<.05; Statistics for data with missing values imputed.

The structure variable which captures the use of human resources is significant in Model 2, where nurse-based models are significantly and positively correlated with satisfaction. In model 3 however, the correlation is still positive, but not significant anymore. The other resource variable equipment is equally positively associated with service satisfaction, and is significant in both Models 2 and 3.

In Model 3, several process variables make contributions to explain satisfaction. Comprehensiveness of consultation, responsiveness, and timeliness are significantly positively associated with satisfaction; Waiting time and empathy are significantly negatively associated.

### Relationships between structures and processes with quality of life

The regression models in Table 3 examine the relationships between quality of life and context, structure, and process variables. Model 1 which includes context variables only explains 17% of variation in EQ5D. Explained variance in quality of life significantly increases 3% from Model 1 to Model 2, which additionally includes structure variables (change R^2^ = .03-.04, p<.001). From Model 2 to Model 3, which adds process variables, the change in explained variance of 2% is again highly significant (change R^2^ = .02-.03, p<.001). Model 3 explains 23% of variance in EQ5D.

**Table 3:** Regression analysis of EQ-5D ^a^.

		Model 1	Model 2	Model 3
		β	β	β
**Covariates**	Age	-.002*	-.002*	-.002*
	Gender			
	- Female (reference)	0	0	0
	- Male	.071*	.075*	.073*
	Education			
	- Minimum school leaving age (reference)	0	0	0
	- More than minimum school leaving age	.038*	.023	.019
	Time since diagnosis	-.002*	-.002*	-.002*
	Stage of diabetes			
	- Lifestyle segment b	0	0	0
	- Medication segment c	-.053*	-.048*	-.045*
	- Insulin injection segment d	-.093*	-.089*	-.084*
	Drink			
	- Non-drinker (reference)	0	0	0
	- Drinker	.062*	.047*	.040*
	Smoke			
	- Former smoker/non-smoker (reference)	0	0	0
	- Current smoker	-.047*	-.043*	-.042*
	Physical activity	.004*	.004*	.003*
	Knowledge of A1c			
	- Unknown value of A1c (reference)	0	0	0
	- Known value of A1c	.033*	.042*	.042*
**Structure**	Human resource model			
	- GP/internist model (reference)		0	0
	- GP/family doctor model		.040	.030
	- Nurse-based model		.049*	.005
	Equipment		.014*	-.002
	Travelling distance to facility		.002*	.002
	Travelling time to facility		-.003*	-.002*
**Process**	Number of follow up visits			-.006*
	Comprehensiveness of follow up visits			.007
	Waiting time in facility			-.001*
	Timeliness			.000
	Responsiveness			.026*
	Empathy			-.019*
	Caring			-.001
	Communication			.009
	R^2^ Change		.03-.04	.02-.03
	R^2^	0.17	.20-.21	0.23
	F Change	24.26–25.74	9.16–11.33	4.37–5.84
	df1	10	5	8
	df2	1206	1219	1214
	Sig of F change	0.000	0.000	0.000

^a^ EQ-5D is measured with 1 utility of full health and 0 utility of death. Unstandardized coefficients,

*p<.05.

All context variables remain significantly associated with quality of life throughout the 3 models, except for education which ceases to be significant in Model 2. The reader may notice that quality of life is negatively associated with the progression over the three stages of the disease included in the analysis.

Let us now consider how EQ5D varies with the structure variables. The nurse-based model is associated with a higher quality of life in Model 2, but not in Model 3. All other structure variables added in Model 2, i.e. equipment, travel distance and travel time relate significantly to EQ5D in Model 2. However, after adding process variables to the analysis in Model 3, only travel time to service remains significantly, and negatively, correlated to EQ5D.

Model 3 reveals that the process variables number of visits, waiting time, and empathy relate significantly and negatively to EQ5D. Responsiveness is the only process variable that is significantly and positively associated with EQ5D.

### Relationships between processes and effective control percentage

As data for effective coverage is collected at network level, the relationships with structure and process variables cannot be analyzed with the regression models used for the patient level reported outcomes service satisfaction and quality of life. Hence, below we briefly present a graphical analysis that sheds lights on the direction of the relationships presented in Fig 1. More specifically, we consider average annual hours of care per patient and average annual costs per patient in relation to effective control percentage.

**Fig 2:**
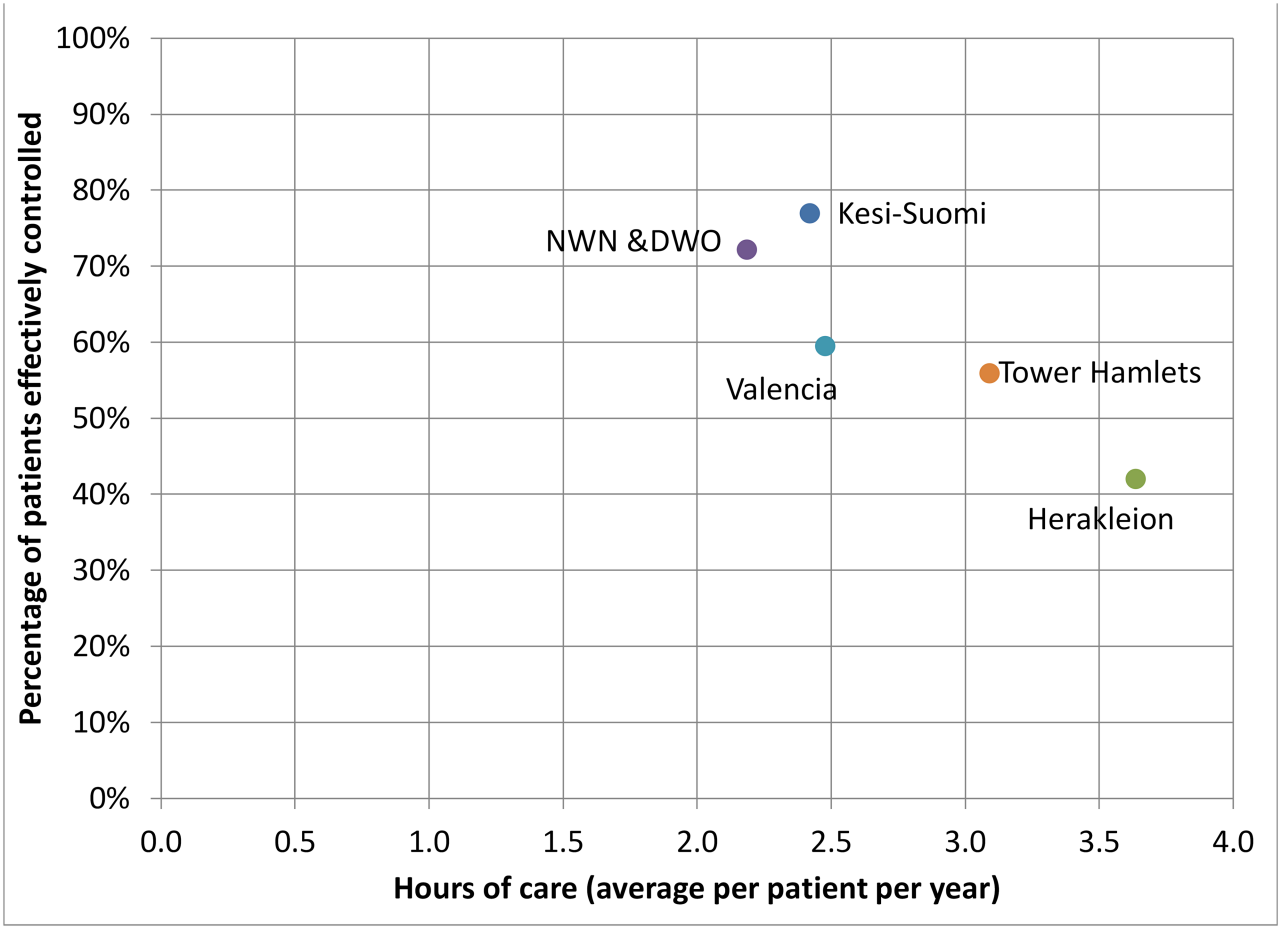
Relationships between total hours of care and the percentage of patients with effective coverage of diabetes care.

**Fig 3:**
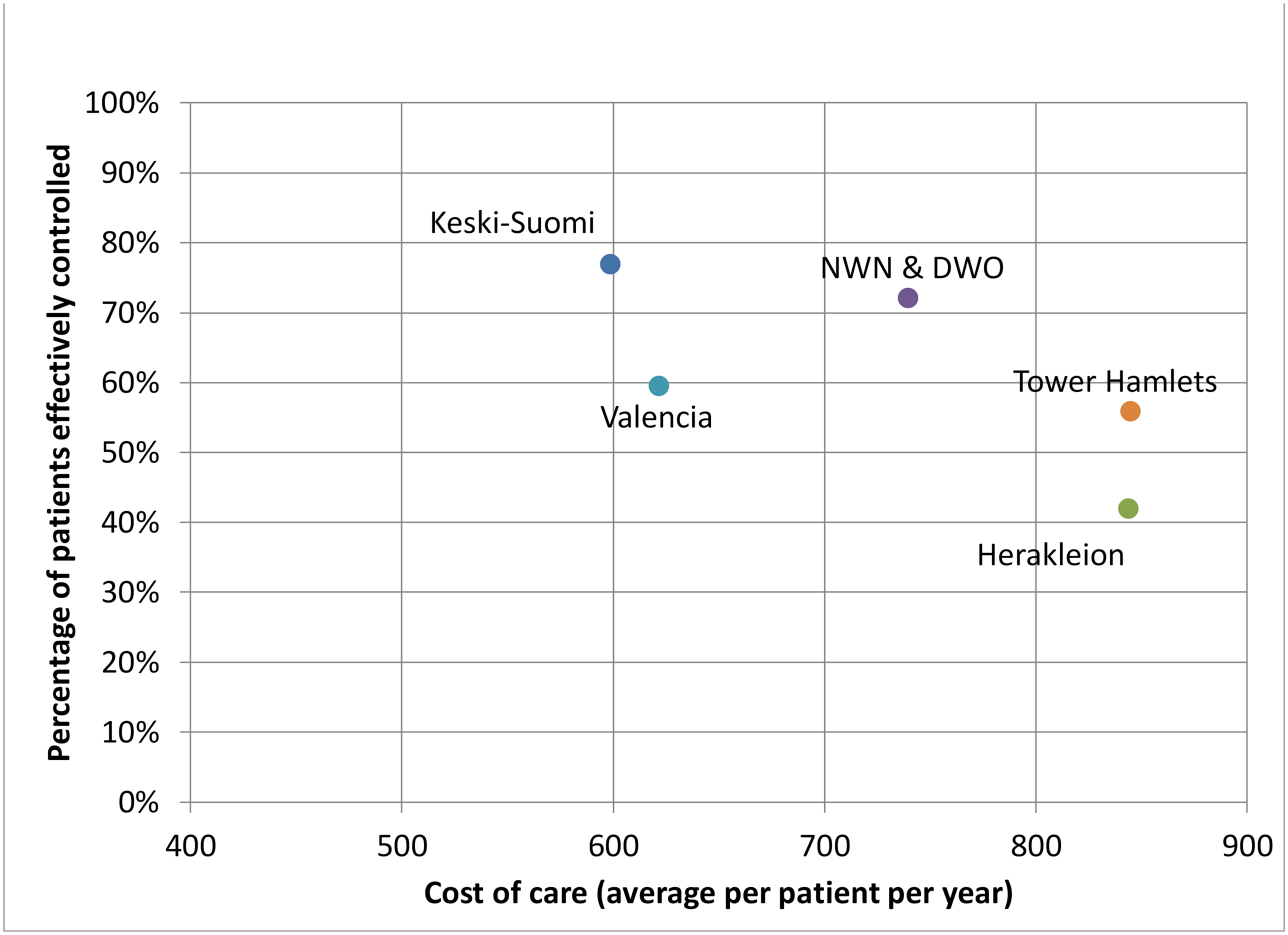
Relationships between costs and the percentage of patients with effective coverage of diabetes care.

Fig 2 indicates that more hours of service provisioning appears to be associated with a lower effective control percentage. Likewise, Fig 3 suggests that lower annual costs per patient are associated with a lower effective coverage percentage. For instance, region Keski-Suomi has the lowest average annual costs per patient and the highest effective coverage percentage. The interpretation of these results is left for the discussion.

## Discussion

We investigated how differences in T2D outcomes relate to differences in context, structure and process in European regional provider networks. The mostly demographic context variables play a modest role in explaining differences in service satisfaction (3%) among the patients in these networks. However, they explain 17% of variation in quality of life among patients. Rather than further analyzing these contextual factors, we focus the discussion on structure and process variables in relation to outcomes. Understanding of these relationships may be particularly instrumental to devising health service interventions to improve effectiveness of T2D networks. Our results provide an initial evidence base on these relationships, and together with the proposed operational model can serve to advancing scientific understanding on T2D networks.

Our findings clearly suggest that the evidence-based structure and process variables included in the analysis are associated with satisfaction. Whereas context variables explained a mere 3% of variation in service satisfaction, adding structures and processes variables resulted in an explained variance in service satisfaction of 44%. Nurse-based models are associated with increased satisfaction, although this relationship ceased to be significant when process variables are included in the model. More generally, the variance explained by human resource models (as adopted by regions) can never exceed the variance explained by the more refined model in which the regions themselves are explicitly included. As the latter models improve only 2% in explained variance over the human resource models (see S5 Appendix), the human resource model adopted by regions appears to be a particularly relevant structure element. In the words of Donadedian, structures which use the nurse-based model appear more likely to induce effective processes.

Of the process variables, the largest contributions are attributed to comprehensiveness of services and the SERVQUAL dimensions responsiveness and timeliness (see also [37]). Interestingly, the SERVQUAL dimension empathy is significantly negatively associated with service satisfaction. Perhaps empathy is perceived stronger by patients whose experiences on other dimensions are worse, and therefore particularly befalls already dissatisfied patients. Our results on the associations of SERVQUAL items with service satisfaction provide first evidence for their relevance in the context of T2D networks.

The number of follow-up visits and therefore health service use was not related to satisfaction, which confirms single provider evidence [38]. The analysis also reveals a significant negative association between waiting time and service satisfaction which partially supports previously reported single provider evidence [39].

Context variables explain 16% out of a total 23% of explained variance in quality of life. Structure and process variables together explain only a modest 6% of variation in quality of life. As was the case for service satisfaction, nurse based models are significantly associated with quality of life only when process variables are not taken into account. The only structure variable that is significant in the final model is travel distance: longer distances to facilities are associated with lower quality of life.

Except for responsiveness, all significant process variables are negatively associated with quality of life. Such a negative relationship is intuitively appealing for waiting time. For empathy, the results are similar to the results found for service satisfaction. The significant negative correlation for hours of care however provide further indications of the mechanism that poor outcomes result in more frequent and more empathic service provisioning. This evidence therefore supports the bidirectional relationship between processes and outcomes depicted in Fig 1, which forms an extensions of the original SPO model [10].

The results on the outcome variable effective coverage percentage suggest a negative relationship with process variables hours of care and cost of services (as further confirmed by the regression analysis presented in S5 Appendix, in which variance in the patient level variable number of visits is significantly and negatively associated with the network level outcome variable effective control percentage). The interpretation that more service provisioning and higher costs cause poorer A1c control seems unrealistic. These relationships may rather indicate again that poor outcomes result in higher service use and cost [40, 41], providing further evidence in support of the SPO model extension. Put differently, the evidence supports Juran’s classic ‘Cost of Quality’ theory [42] which posits that improving quality reduces cost.

While aware that the cross-sectional study design does not allow us to establish causality of relationships, our findings provide suggestions for improving service satisfaction in T2D networks. Increasing the role of nurses is likely to improve outcomes, either directly or via process improvements. As T2D prevalence rises, building the volume and skill set of nurses may be easier and less costly than increasing the number of physicians. In general, however, our results suggest targeting process interventions rather than structure interventions, as the process variables are more significantly associated with outcomes.

Let it be noted however that we have investigated independent relationships of the structure and process variables with the outcomes, an alternative assessment approach which also considers joint effects of structure and process variables may reveal further insights and alternative improvement strategies.

## Conclusions

Our results provide first evidence for T2D networks in support of Donabedian’s SPO model, which argues that better structures and better processes are likely to improve outcomes. The evidence found is stronger for experiential outcomes than for functional and clinical outcomes. This may in part be due to the complex, bidirectional, nature of the relations that processes have with clinical and functional outcomes.

The standardized operational models used in this research have successfully enabled to capture network structure and process variables, and can be applied and developed further to expand the presented initial evidence base. To advance this evidence base and—more generally—understanding of the relationships between structures and processes with outcomes in T2D provider network, we recommend controlled experiments in which all outcome data, including A1c data, are collected at the patient level.

## Supporting information

S1 Framework Framework for description of service structure, process, behavior, and outcome. (DOCX)

S1 Appendix Background information of study regions. (DOCX)

S2 Appendix Instruments, ethical approval, and data collection procedures. (DOCX)

S3 Appendix Operational model templates and description of services for Netherlands case study. (XLS)

S4 Appendix Synthesis of data for study regions. (XLSX)

S5 Appendix Additional analysis with regions. (DOCX)
