# Correction: The relationship between context, structure, and processes with outcomes of 6 regional diabetes networks in Europe

**DOI:** 10.1371/journal.pone.0225619

**Published:** 2019-11-18

**Authors:** Mahdi Mahdavi, Jan Vissers, Sylvia Elkhuizen, Mattees van Dijk, Antero Vanhala, Eleftheria Karampli, Raquel Faubel, Paul Forte, Elena Coroian, Joris van de Klundert

There are multiple errors in this article [1]. The authors provide the following updates in this Correction.

The authors have agreed to have Mahdi Mahdavi serve as the corresponding author for this article [1] instead of Jan Vissers. Any inquiries should be directed to Dr. Mahdavi at National Institute of Health Research (NIHR), Tehran University of Medical Sciences, Tehran, I. R. Iran (* Email: info.mahdavi@gmail.com).

The authors would like to clarify that Joris van de Klundert's primary affiliation when contributing to this project was with the Prince Mohammad bin Salman School for Business and Entrepreneurship, King Abdullah Economic City, Saudi Arabia. Dr. van de Klundert is also affiliated with the Institute of Health Policy and Management, Erasmus University Rotterdam, as noted in [1].

The Data Availability statement for this paper is incorrect. The correct statement is:

The operations management data as well as patient aggregate data from the Managed Outcomes consortium used in this research are provided as Supporting Information (S1 Appendix-S5 Appendix). Due to shared nature of patient survey data, that underlie our findings, no single institution is entitled to share the entire dataset. Each partner institution owns data collected in its country, which can be made available upon request by contacting Dr. Paulus Torkki (paulus.torkki@helsinki.fi), Dr. Eleftheria Karampli (ekarabli@esdy.edu.gr), Ms. Elena Coroian (elena.coroian@ili.fau.de), Dr. Sylvia Elkhuizen (elkhuizen@eshpm.eur.nl), Dr. Raquel Faubel (raquel.faubel@uv.es), and Dr. Paul Forte (Paul.forte@balanceofcare.com) for data collected in Finland, Greece, Germany, The Netherlands, Spain, and the United Kingdom, respectively.

The following statement is hereby added to the Acknowledgments:

In particular we thank Uwe Konerding who developed the questionnaire used for data collection, supervised the survey in all study countries, and provided detailed comments on earlier drafts of this paper.

The following sentences should include citations for Konerding U, Bowen T, Elkhuizen SG, Faubel R, Forte P, Karampli E, et al. (2017) The impact of travel distance, travel time and waiting time on health-related quality of life of diabetes patients: An investigation in six European countries. Diabetes Res Clin Pract. 126:16–24. https://doi.org/10.1016/j.diabres.2017.01.014. Epub 2017 Feb 2.

- F*irst sentence of the third paragraph of the Introduction*: Previous studies have indeed shown that these outcomes vary among T2D networks [6–9].
- L*ast sentence of the fifth paragraph of the Introduction*: Moreover, structures which improve access to care have been reported to positively affect service satisfaction and the health state of T2D patients [15, 16].

Additionally, in the Variables, Outcomes subsection of the Materials and methods, the following sentence should be inserted as the *third sentence of the second paragraph*:

A study performed with the same data as analysed here has given evidence for the validity of the items addressing these dimensions (Konerding et al. 2014).

The reference above is: Konerding U, Elkhuizen SG, Faubel R, Forte P, Malmström T, Pavi E, et al. (2014) The validity of the EQ-5D-3L items: an investigation with type 2 diabetes patients from six European countries. Health Qual Life Outcomes. 12:181. https://doi.org/10.1186/s12955-014-0181-5.

The authors apologize for these errors in the published article.
