# Supplementary material for: The relationship between context, structure, and processes with outcomes of 6 regional diabetes networks in Europe
Source: PLoS One. 2018 Feb 15;13(2):e0192599. doi: 10.1371/journal.pone.0192599 (PMC5813938; doi:10.1371/journal.pone.0192599)
Supplement: S1 Framework — (DOCX) [file pone.0192599.s001.docx]

**Framework for description of service structure, process, behavior, and outcome**

| ***Abstraction level*** | | ***Description*** |
| --- | --- | --- |
| **Demand** | Health Service User | Service user refers to the individual patient who demands health services. Service user is defined with regard to demographic characteristics, disease history, and disease—specific medical conditions requiring the health services. |
|  | Demand Segment | Segments refer to mutually exclusive subsets of the population of health service users with a common demand for health services (e.g. because of sharing a same health condition). |
|  | Demand Location | Locations define areas within the geographical areas which are meaningful to distinguish because of differences in demand and or geographical properties |
| **Services/process** | Service element | A service element is the atomic unit of service. |
|  |  | For each service element the resource requirements specify the type of resources (see below) required to perform the service element, as well as the expected usage of each of these types (e.g. in hours). |
|  |  | A service element can be described in terms of waiting times, frequency, length of stay, transitions to another service element and cost. |
|  |  | The costs of a service element are defined as the sum of the costs of the required resource usages. |
|  | Service journey | A service journey consists of a partially ordered set of service elements, which are provided to health service users from a demand segment. |
|  |  | Operational and financial performances of a service journey are aggregated from corresponding service elements performance. |
|  |  | The costs of a service journey are defined as the sum of the costs of the service elements involved. Costs are estimated per patient per year etc. |
|  |  | Transition probability refers to the distribution of health service users from the demand segment corresponding to the service journey over possible succeeding demand segments (and corresponding service journeys). |
|  | Service user journey | User journey refers to the sequence of services that a health service user follows (defined through the sequence of service journeys). |
|  |  | The costs of a service user journey consist of the sum of the costs of the service journeys involved. |
| **Structure** | Resource | A resource is a means to provide a service. Resources are described according to their type, availability, capacity and unit cost. |
|  |  | With regard to type, resources are distinguished into devices, facilities, and human resources. |
|  |  | Resource availability refers to the amount of resources which is available to deliver services per time period. |
|  |  | Resource capacity refers to the amount of health service users that can be treated in a time period. |
|  |  | Resource cost refers to the monetary cost of a resource per unit (e.g. per hour). |
|  | Service Provision Point | Provision point refers to a location where resources required to provide a service are located. |
|  |  | Access to provision point is measured by physical distance of and travel time from the demand location of the health service user to the provision point. |
|  | Service provider | A health service provider is a person or a legal entity who/which delivers health services to patients. |
| **Behaviour** | General health related behaviour | General health behaviour refers to the life style of the health service user, such as smoking, diet, and physical exercise behaviour |
|  | Service related behaviour | Service related behaviour refers to behaviour which directly relates to the health services, e.g. treatment adherence or follow-up to advices by service provider. |
| **Outcome** | Health outcomes | Health outcomes are features of the health care user’s health. A variety of quite different health outcomes can be considered ranging from perceived health related quality of life as reported by the health service user to specific clinical outcomes as reported by the health care provider. |
|  | Service outcomes | Service outcomes regards both provider measures on service performance (such as waiting times) as well as health service users perceptions of service provisioning, and the valuation of the service provisioning by health service users. |
