# Supplementary material for: The relationship between context, structure, and processes with outcomes of 6 regional diabetes networks in Europe
Source: PLoS One. 2018 Feb 15;13(2):e0192599. doi: 10.1371/journal.pone.0192599 (PMC5813938; doi:10.1371/journal.pone.0192599)
Supplement: S1 Appendix — (DOCX) [file pone.0192599.s002.docx]

**Background information of study regions**

The regions where provider networks were investigated are Keski-Suomi (Finland), Bamberg (Germany), Herakleion (Greece), NieuweWaterwegNoord & DelftWestlandOostland (NWN & DWO) (The Netherlands), Valencia (Spain), and Tower Hamlets (United Kingdoms).

 Demographic information and important contextual factors of health care system and current diabetes practice are described below:

**Keski-Suomi (Finland)**

Keski-Suomi lies in the center of Finland and covers 19.950 sq km. The population of the Keski-Suomi counts 272,784 inhabitants. The population density is 13,7 per sq km. The region of Keski-Suomi is comprised of 23 municipalities which vary largely in geographical size and population.

 Finland has a national health services system with a strong role of municipalities for organising health services for their residents and with gate-keeping arrangements for access to care. Diabetes care is primarily delivered in primary care centers by general practitioners and practice nurses or diabetes nurses. The focus in diabetes treatment is on life style and early intervention.

**Bamberg (Germany)**

The region is located in the south-east of Germany. It has 144,211 inhabitants (2010) and covers 1,168 km²; the population density 123,5/km². The administration of Bamberg region is seated in Bamberg, which is a municipality not associated with a county.

 Germany has a social health insurance based health care system with many market mechanisms, a free access to care and free choice of medical doctors. Diabetes care is primarily delivered by general practitioners. There is no national approach for the primary prevention of diabetes. The emphasis in diabetes treatment is on medication-based treatment of patients who already suffer from diabetes.

**Herakleion (Greece)**

The Regional Unit of Herakleion (RUH) is located in the central-east part of the island of Crete and has a size of 2641 sq km. The population counts 304,270; the population density is 115,2 per sq km. The city of Herakleion is the Regional Unit’s capital with 173,450 residents (57% of the RUH population). During 1991-2011 the population of the RUH has increased by 38,562 inhabitants (15%), while available data by age groups indicate an ageing trend.

Greece has a mixed healthcare system in which a national health services system (ESY) and a social insurance system co-exist. During the study period, primary healthcare services in Greece were provided by: hospital outpatient departments and health centers operating in the National Health System (ESY), healthcare facilities owned by Social Insurance Funds, health centres owned by local authorities (municipalities) and private sector healthcare providers (predominantly physicians in solo private practices who contract with social insurance funds). The primary healthcare system is currently under reform. Diabetes care is delivered by medical specialists (e.g. internists) and general practitioners.

**Valencia (Spain)**

The Region of Valencia (East Spain) is located in central and south-eastern Iberian Peninsula. The region of Valencia covers 23,259 km² of Spain with 5.02 million inhabitants in 2008. The part of the city of Valencia serviced by the provider network investigated, counts 262.191 inhabitants; the area is 173 sq km; the population density is 6047 inhabitants per sq km..

The statutory SNS is universal coverage-wise funded from taxes based on the public sector. Provision is free of charge at the point of delivery with the exception of the pharmaceuticals prescribed (co-payment). Health competences were totally devolved to the regional level in 2002. A regional health system consists of a regional ministry in charge of policies, regulation and planning, and a regional health service performing as provider. Diabetes care is primarily delivered by primary healthcare system with a specific protocol guide. Primary care is assisted where necessary through the Specialized Care (Diabetes Units). The focus in the diabetes treatment is on early diagnosis, lifestyle strategies and avoiding complications

**Nieuwe Waterweg Noord & Delfland Westland Oostland (NWN & DWO) (The Netherlands)**

NWN & DWO is situated in the western part of the Netherlands, between Rotterdam and The Hague. The number of inhabitants is 330,464; the region size is 273 sq km; the population density is 1624 inhabitants per sq km. The region consists of 2 sub-regions, each consisting of a number of municipalities: The region Nieuwe Waterweg Noord (NWN) consists of Delft, Westland, Midden Delfland, and Pijnacker-Nootdorp. The region Delfland, Westland, Oostland (DWO) consists of Schiedam, Vlaardingen. Maassluis and Hoek van Holland (sub-community of Rotterdam).

 The Netherlands has a social health insurance based health care system, with regulated market mechanisms and gate-keeper arrangements. Diabetes care is primarily provided by general practitioners, practice nurses and district nurses. The emphasis in diabetes treatment is on life style, early intervention and avoiding complications.

**Tower Hamlets, London (UK)**

The London Borough (LB) of Tower Hamlets is in inner London, immediately due east of the City of London financial district and bordering the north side of the river Thames. It covers an area of 20.7 km2 and, with a population (mid-2010 estimate) of 238,100, it is one of London’s smallest and most densely populated Boroughs (population density: 11.502 per sq km). The demographic structure of LB Tower Hamlets is young with 19% of the population under 15 years of age (compared with 17% for the rest of the UK). By contrast, the proportion of people over-65 is only 7% compared with the UK average of 16.5%. 60% of those under 20 are Bangladeshi, with 60-70% of those 20 and above are white. This proportion generally increases in the older age bands (over 80% of over 80s are white). Over the next five years, the proportions of different ethnicities are not expected to change significantly.

The UK health care system is a centrally-funded national health services system with non-emergency access to secondary care only through primary care-based general practitioners (family doctors). Long-term diabetes care is delivered in the primary care setting team mainly by general practitioners and community nurses (some of whom have specialist diabetes qualifications). The focus in diabetes care is on controlling blood sugar levels by maintaining a balance in the patient's clinical indicators (HBA1C, cholesterol, blood pressure) rather than focusing specifically on disease stages.
