# Supplementary material for: The relationship between context, structure, and processes with outcomes of 6 regional diabetes networks in Europe
Source: PLoS One. 2018 Feb 15;13(2):e0192599. doi: 10.1371/journal.pone.0192599 (PMC5813938; doi:10.1371/journal.pone.0192599)
Supplement: S2 Appendix — (DOCX) [file pone.0192599.s003.docx]

## Instruments, ethical approval, and data collection

The diabetes model presents a comprehensive set of variables for analysis of T2D operations and outcomes. We used three sources to collect data for operations, behaviour, and outcomes: medical information systems and patient records; patient questionnaires and interviews with local staff. Below we describe the data collection procedures and instruments.

Operations (service and structure) data for each regional instance was collected by interview requests for data from existing local information systems. A standardized spreadsheet model was developed which captured the diabetes model and corresponding calculations.

Data on demand characteristics, behaviour, and outcomes was collected by approaching type 2 diabetes patients living in the regions via a survey which, amongst other things, asked for age, gender, education, time since diagnosis, and diabetes stage (demand segment) of participants. Patients in order to be included in the survey must be treated only by primary care networks or non-hospital based settings.

Samples of T2D patients studied in Bamberg, Herakleion, and Valencia were surveyed in the second half of 2011 and in the other regions in the first half of 2012. An appropriate sample of institutions in each network (e.g. health centre or general practitioner surgery) was chosen to participate in the survey.

The percentage of patients with HbA1c<53 mmol/mol was collected from provider information systems and patient records, but the HbA1c level was also asked for in the survey. Data on complications was collected from provider information systems and patient records, but also addressed in the survey. The EuroQol instrument (EQ-5D-3L) (‘five dimensions’)) which had validity and reliability across all regions was used to measure the perception of the patients of their health conditions [41]. The perception of service quality was measured by a short, generic version of the SERVQUAL instrument, which includes six aspects of quality: tangibles, timeliness, responsiveness, empathy, caring and communication (Bowers, Swan, & Koehler, 1994). Satisfaction with services was captured by a single question which measured satisfaction on a scale from ‘extremely dissatisfied’ to ‘extremely satisfied’. The patient survey also sought data on the perceived aspects of operations: traveling time to and distance from the main provider offices or clinics; waiting time in the provider office or clinic; frequency of visits in a one year period; and the patient’s perception of the comprehensiveness of consultation.

The study was approved in each country involved in the study. The Keski Suomi study was approved by the Ethics Committee of the Central Finland Health Care District. The Bamberg study was approved by the Ethics Committee of the Medical Faculty of the Friedrich-Alexander University in Erlangen-Nürnberg. The Herakleion study was approved by the Scientific Committee of the hospital in Herakleion. The NieuweWaterwegNoord & DelftWestlandOostland study was approved by the board of directors of the Primary Care Group ZEL. The Valencia study was approved by the Hospital La Fe Ethical Committee. The Tower Hamlets study was approved by the NHS National Research Ethics Service. Permission for use of data was received from the Ethics Committee of the Central Finland Health Care District (statistical data at aggregate level), the Ethics Committee of the Medical Faculty of the Friedrich-Alexander University in Erlangen-Nürnberg (statistical data at aggregate level), the Scientific Committee of the hospital in Herakleion (statistical data and access to patient records), the Scientific Council of the IPCI system of the department of Medical Information of the Erasmus Medical Centre (statistical data at aggregate level), the Hospital La Fe Ethical Committee (statistical data at aggregate level) and the NHS National Research Ethics Service (statistical data and access of patient records through the clinicians of the local diabetes research network).

The recruitment of participants was constrained by several limitations defined by the health care providers investigated. Moreover, several different statistical tests with different properties were intended to be used. So an exact a-priori determination of the sample size is neither of much use nor possible. However, to get a hint as to which sample size should at least be aspired we determined the sample size which would be needed for an ANOVA performed for the six different regions to be compared. The sample size was determined for a small effect size (f = 0.10) [68], **α** equal to 0.05, and statistical power equal to 0.80 (Field, 2009). Based on these assumptions, total sample size was 1284 patients.

The questionnaire was developed in English and then translated into the native language of each region (Sperber, 2004). Two native speakers of the target region’s language translated it from English, and one native English speaker translated it back from a target language to English to ensure its validity. The questionnaires were also culturally adapted where necessary.

On behalf of the Managed Outcomes project, providers with a right to access patient information sent questionnaires to patients. The questionnaire also included research information and an invitation to participate in this research. Completion of the survey by patients was entirely voluntary. All personal patient identifiers were removed or disguised so the respondent was not identifiable either through the measurements they provided or through any free text provided for the open questions. As there was no patient identifiable data on the survey form it was not possible to follow-up any non-respondents.

The questionnaire was given to the patients by their GP or primary care team member with whom the user was registered. In Herakleion questionnaires were mailed to patient addresses. In total 5,972 patients in 31 health facilities across all regions were approached. One thousand six hundred and thirty eight questionnaires were returned, i.e. there was a response rate of 27.4%.

Accordingly, for the regression analyses, pooled unstandardised regression coefficients and the corresponding tests are based upon the corresponding pooled standard errors. For the other relevant statistics no adequate procedure for pooling over the different completed data sets are known at the present. So, for these statistics, different variants were reported: on the one hand the statistics for the data set comprising only cases without missing data and on the other hand the ranges of the statistics for the five different completed data sets. As long as data are missing completely at random inferential tests performed only for the complete data are more conservative as a true test would be whereas the tests performed for the completed data are more liberal. So the true statistical test result can be assumed between both of them.

References

Bowers, M. R., Swan, J. E., & Koehler, W. F. (1994). What attributes determine quality and satisfaction with health care delivery? *Health Care Management Review*, *19*(4), 49–55.

Field, A. (2009). *Discovering statistics using SPSS*. Sage Publications Limited.

Sperber, A. D. (2004). Translation and validation of study instruments for cross-cultural research. *Gastroenterology*, *126*, S124–S128. https://doi.org/10.1053/j.gastro.2003.10.016
