# Supplementary material for: The relationship between context, structure, and processes with outcomes of 6 regional diabetes networks in Europe
Source: PLoS One. 2018 Feb 15;13(2):e0192599. doi: 10.1371/journal.pone.0192599 (PMC5813938; doi:10.1371/journal.pone.0192599)
Supplement: S5 Appendix — (DOCX) [file pone.0192599.s006.docx]

**Appendix S 5: Additional analysis with regions**

Regression analysis of service satisfaction ^a^

|  |  | Model 1  β | Model 2  β | Model 3  β |
| --- | --- | --- | --- | --- |
| Covariates | Age | 0.023 | -0.015 | 0.003 |
|  | Gender ^b^ | 2.849 | 4.407* | 3.790* |
|  | Education ^c^ | 4.582* | 1.300 | 0.463 |
|  | Time since diagnosis | -0.003 | -0.026 | -0.041 |
|  | Lifestyle segment (reference) ^b^ |  |  |  |
|  | Medication segment ^c^ | -0.652 | 0.269 | -0.985 |
|  | Insulin injection segment ^d^ | 0.071 | 0.016 | -2.400 |
|  | Drink ^d^ | 2.105 | -0.232 | -1.148 |
|  | Smoking ^e^ | -3.420 | -3.394 | -3.051 |
|  | Physical activity | 0.200 | 0.177 | 0.061 |
|  | Knowledge of HBA1c ^f^ | -0.752 | 0.507 | -0.332 |
| Structures | UK (reference) |  |  |  |
|  | Finland |  | 2.679 | 3.311 |
|  | Germany |  | -2.894 | -1.644 |
|  | Greece |  | -3.367 | -1.150 |
|  | The Netherlands |  | 6.998* | 2.589 |
|  | Spain |  | -7.634* | -2.961 |
|  | Up to date equipment |  | 7.485* | 2.286* |
|  | Travelling distance to facility |  | -0.024 | -0.051 |
|  | Travelling time to facility |  | 0.122 | 0.126 |
| Processes | Number of follow up visits |  |  | 0.142 |
|  | Comprehensiveness of follow up visits |  |  | 2.330* |
|  | Waiting time in facility |  |  | -0.095* |
|  | Timeliness |  |  | 1.666* |
|  | Responsiveness |  |  | 3.040* |
|  | Empathy |  |  | -1.702* |
|  | Caring |  |  | 3.220* |
|  | Communication |  |  | 0.794 |
|  |  |  |  |  |
|  | R^2^ Change |  | .28-.29 | .11-.13 |
|  | R^2^ | .02-.03 | .30-.32 | .42-.44 |
|  | F Change | 2.90-3.86 | 61.17-66.67 | 30.15-36.03 |
|  | df1 | 10 | 8 | 8 |
|  | df2 | 1247 | 1239 | 1231 |
|  | Sig of F change | 0.001 | .000 | .000 |

^a^ Service satisfaction measured on a scale ranging from 0 to 100. Human resource models are replaced by regions. Unstandardized coefficients, *p<.05. Statistics for data with missing values imputed. ^b^ 0=female, 1= male. ^c^ 0= minimum school leaving age, 1= more than minimum school leaving age. ^d^ 0= No alcohol consumption, 1= alcohol consumption. ^e^ 0= Non-smoker and former smoker, 1= smoker. ^f^ 0= HbA1c not known, 1= HbA1c known.

Hierarchical regression analysis of quality of life ^a^

|  |  | Model 1  β | Model 2  β | Model 3  β |
| --- | --- | --- | --- | --- |
| Covariates | Age | -0.002* | -0.003* | -0.003* |
|  | Gender ^b^ | 0.071* | 0.079* | 0.076* |
|  | Education ^c^ | 0.038* | 0.008 | 0.008 |
|  | Time since diagnosis | -0.002* | -0.002* | -0.002* |
|  | Lifestyle segment (reference) ^b^ |  |  |  |
|  | Medication segment ^c^ | -0.053* | -0.054* | -0.052* |
|  | Insulin injection segment ^d^ | -0.093* | -0.095* | -0.091* |
|  | Drink ^d^ | 0.062* | 0.044* | 0.04* |
|  | Smoking ^e^ | -0.047* | -0.039* | -0.037* |
|  | Physical activity | 0.004* | 0.003* | 0.003* |
|  | Knowledge of HBA1c ^f^ | 0.033* | 0.041* | 0.041* |
| Structures | UK (reference) |  | 0 | 0 |
|  | Finland |  | 0.097* | 0.093* |
|  | Germany |  | 0.051* | 0.072* |
|  | Greece |  | 0.016 | 0.053* |
|  | The Netherlands |  | 0.1* | 0.09* |
|  | Spain |  | 0.082* | 0.116* |
|  | Up to date equipment |  | 0.014* | -0.001 |
|  | Travelling distance to facility |  | 0.001 | 0 |
|  | Travelling time to facility |  | -0.002* | -0.002* |
| Processes | Number of follow up visits |  |  | -0.007* |
|  | Comprehensiveness of follow up visits |  |  | 0.005 |
|  | Waiting time in facility |  |  | -0.001* |
|  | Timeliness |  |  | 0.002 |
|  | Responsiveness |  |  | 0.023* |
|  | Empathy |  |  | -0.021* |
|  | Caring |  |  | 0 |
|  | Communication |  |  | 0.011 |
|  |  |  |  |  |
|  | R^2^ Change |  | 0.06 | .02-.03 |
|  | R^2^ | 0.17 | 0.23 | .25-.26 |
|  | F Change | 24.26-25.74 | 11.13-12.16 | 3.87-5.43 |
|  | df1 | 10 | 8 | 8 |
|  | df2 | 1219 | 1211 | 1203 |
|  | Sig of F change | 0.000 | 0 | 0 |

^a^ EQ-5D is measured with 1 utility of full health and 0 utility of death. Human resource models are replaced by regions. Unstandardized coefficients, *p<.05. Statistics for data with missing values imputed. ^b^ 0=female, 1= male. ^c^ 0= minimum school leaving age, 1= more than minimum school leaving age. ^d^ 0= No alcohol consumption, 1= alcohol consumption. ^e^ 0= Non-smoker and former smoker, 1= smoker. ^f^ 0= HbA1c not known, 1= HbA1c known.

Relationships between number of visits and effective coverage ^a^

|  |  | Number of visits | |
| --- | --- | --- | --- |
|  | Variables | Model 1  β | Model 2  β |
| 1 | Age | .01 | .00 |
|  | Gender ^b^ | -.06 | .00 |
|  | Education ^c^ | .10 | .17 |
|  | Time since diagnosis | .01 | .01 |
|  | Demand segment 2 | -.58* | -.39 |
|  | Demand segment 3 (reference) |  |  |
|  | Demand segment 4 | .55** | .44* |
| 2 | Good performance in effective coverage ^d^ |  | -.45** |
|  | Average performance in effective coverage ^e^ (reference) |  |  |
|  | Poor performance in effective coverage ^f^ |  | .98** |
| Statistics for data with missing values imputed | |  |  |
|  | R^2^ Change |  | .04-.05 |
|  | R^2^ | .02-.03 | .06-.08 |
|  | F Change | 4.15-5.37 | 18.10-21.57 |
|  | df1 | 6 | 3 |
|  | df2 | 1251 | 1248 |
|  | P value of ∆f | 0.000 | 0.000 |

^a^ Unstandardized coefficients, *p<.05; **p<.01.

^b^ 0=female, 1= male. ^c^ 0= minimum school leaving age, 1= more than minimum school leaving age.

^d^ This group consists of Keski-Suomi and NWN & DWO where the percentage of well-controlled patients exceeds 70 percent. ^e^ This groups is formed by Valencia and Tower Hamlets (70%< percentage of controlled patients <50%). ^f^ Poor control group represents Herakleion, with a lowest percentage of controlled patients (<50%).
